# Nursing home leaders’ and nurses’ experiences of resources, staffing and competence levels and the relation to hospital readmissions – a case study

**DOI:** 10.1186/s12913-018-3769-3

**Published:** 2018-12-12

**Authors:** Malin Knutsen Glette, Olav Røise, Tone Kringeland, Kate Churruca, Jeffrey Braithwaite, Siri Wiig

**Affiliations:** 1 Faculty of Health, Western Norway University of Applied Sciences, Haugesund, Norway; 2 Division of Orthopedic Surgery, Oslo University Hospital, Oslo, Norway; 3 Faculty of Health Sciences, SHARE – Centre for Resilience in Healthcare, University of Stavanger, Stavanger, Norway; 4 Institute of Clinical Medicine, University of Oslo, Oslo, Norway; 5 Centre for Healthcare Resilience and Implementation Science, Australian Institute of Health Innovation, Macquarie University, Sydney, Australia

**Keywords:** Hospital readmissions, Patient safety, Nurse staffing, Nurse competence, Financial resources

## Abstract

**Background:**

Thirty-day hospital readmissions represent an international challenge leading to increased prevalence of adverse events, reduced quality of care and pressure on healthcare service’s resources and finances. There is a need for a broader understanding of hospital readmissions, how they manifest, and how resources in the primary healthcare service may affect hospital readmissions. The aim of the study was to examine how nurses and nursing home leaders experienced the resource situation, staffing and competence level in municipal healthcare services, and if and how they experienced these factors to influence hospital readmissions.

**Method:**

The study was conducted as a comparative case study of two municipalities affiliated with the same hospital, chosen for historical differences in readmission rates. Nurses and leaders from four nursing homes participated in focus groups and interviews. Data were analyzed within and across cases.

**Results:**

The analysis resulted in four common themes, with some variation in each municipality, describing nurses’ and leaders’ experience of the nursing home resource situation, staffing level and competence and their perception of factors affecting hospital readmissions. The nursing home patients were described as becoming increasingly complex with a subsequent need for increased nurse competence. There was variation in competence and staffing between nursing homes, but capacity building was an overall focus. Economic limitations and attempts at saving through cost-cutting were present, but not perceived as affecting patient care and the availability of medical equipment. Several factors such as nurse competence and staffing, physician coverage, and adequate communication and documentation, were recognized as factors affecting hospital readmissions across the municipalities.

**Conclusion:**

Several factors related to nurses’ and leaders’ experience of the resource situation, staffing and competence level were suggested to affect hospital readmissions and the municipalities were similar in their answers regarding these factors. Patients were perceived as more complex with higher patient mortality forcing long-term nursing homes to shift towards an acute care or palliative function, and short-term nursing homes to function as “small hospitals”, requiring higher nurse competence. Staffing, competence and physician coverage did not seem to have adjusted to the new patient group in some nursing homes.

**Electronic supplementary material:**

The online version of this article (10.1186/s12913-018-3769-3) contains supplementary material, which is available to authorized users.

## Background

Thirty-day hospital readmissions are challenging for healthcare services internationally, signalling an increased prevalence of adverse events, and reduced quality of care. They contribute to major strains on healthcare services’ resources and finances [1]. In Norway hospital readmissions have been an increasing problem over the past five years, with readmission rates ranging from 10.3 - 22.9% across different municipalities that provide primary health and social care [2]. Studies show that the incidence of hospital readmissions are higher among elderly patients compared to the general population, and patients with chronic illnesses and comorbidities are overrepresented [3, 4].

As a part of the Coordination Reform introduced to the Norwegian healthcare system in 2012, municipalities were required to take on more of the secondary healthcare services tasks, leading to earlier hospital discharges and more complex patients being discharged to the municipal healthcare services [5]. This has, in turn, led to increased pressure and new demands on nursing homes and homecare services, including the need for staff to perform more complex nursing tasks, and accept higher workloads, especially the amount of administrative work, leading to concomitant work pressures on staff [6].

In parallel with other countries, within the next decade, the proportion of elderly people in Norway will increase, with associated additional public health and economic burdens [7, 8]. Norwegian population projections are predicting an increase from four to 10 % in people aged 80 years or older by 2060 [9]. The aging population will create even more pressure on the resources of healthcare services and may further compound the problem of hospital readmissions. Therefore, it is important to gain a broader understanding of how hospital readmissions manifest and, more specifically, how resources in the municipal healthcare services may affect hospital readmissions.

Most studies of hospital readmissions focus on hospital readmission rates [1, 10], their predictors [3, 11, 12], attempts to reduce them [13, 14] and improvement measures [15, 16]. There has been less qualitative focus on understanding the factors that lead to hospital readmissions from the perspective of leaders and nurses working in primary care. Indeed, primary healthcare providers have mostly been omitted from readmission research, despite their important role in caring for the patient post hospital discharge. Therefore there is a need to investigate hospital readmissions from the perspective of primary healthcare services and to document the issues municipal healthcare personnel perceive as influencing hospital readmissions [25].

### Aim and research question

The aim of the study was to examine how nurses and nursing home leaders experienced the resource situation, staffing and competence level in municipal healthcare services, and if and how they experienced these factors to influence hospital readmissions. The following research question guided the study: *How do nurses and leaders in nursing homes experience the resource situation, staffing and competence level, and how do they consider these factors as possible reasons for hospital readmissions from their organization?*

By illustrating nurses and nursing home leaders’ experiences of nursing home resources and their perception of factors influencing hospital readmissions, this paper provides a broader insight in the hospital readmission problem and how resources may affect hospital readmissions.

## Methods

### Study design

**Fig. 1:**
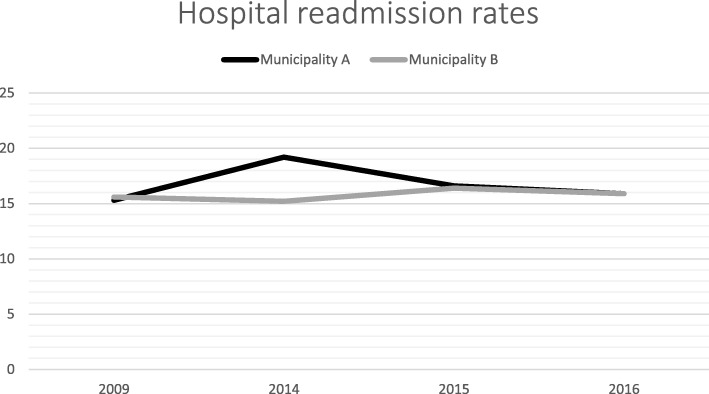
Hospital readmission rates in municipality A and B

This research was conducted as a comparative embedded case study of two Norwegian municipalities. The municipalities were selected for comparison because they were affiliated with the same hospital, and had differences in their readmission rates at the time of recruitment (19.2% in Municipality A and 15.2% in Municipality B in 2014), consequently anticipating contrasting results and variation between the municipalities (contrasting case selection) [17]. However, during the research period and prior to the recruitment of the included participants, the differences between the selected municipalities evened out (Fig. 1.) [2]. Nevertheless, of two cases allowed for exploration of similarities and differences between the included municipalities [17].

### Context

**Fig. 2:**
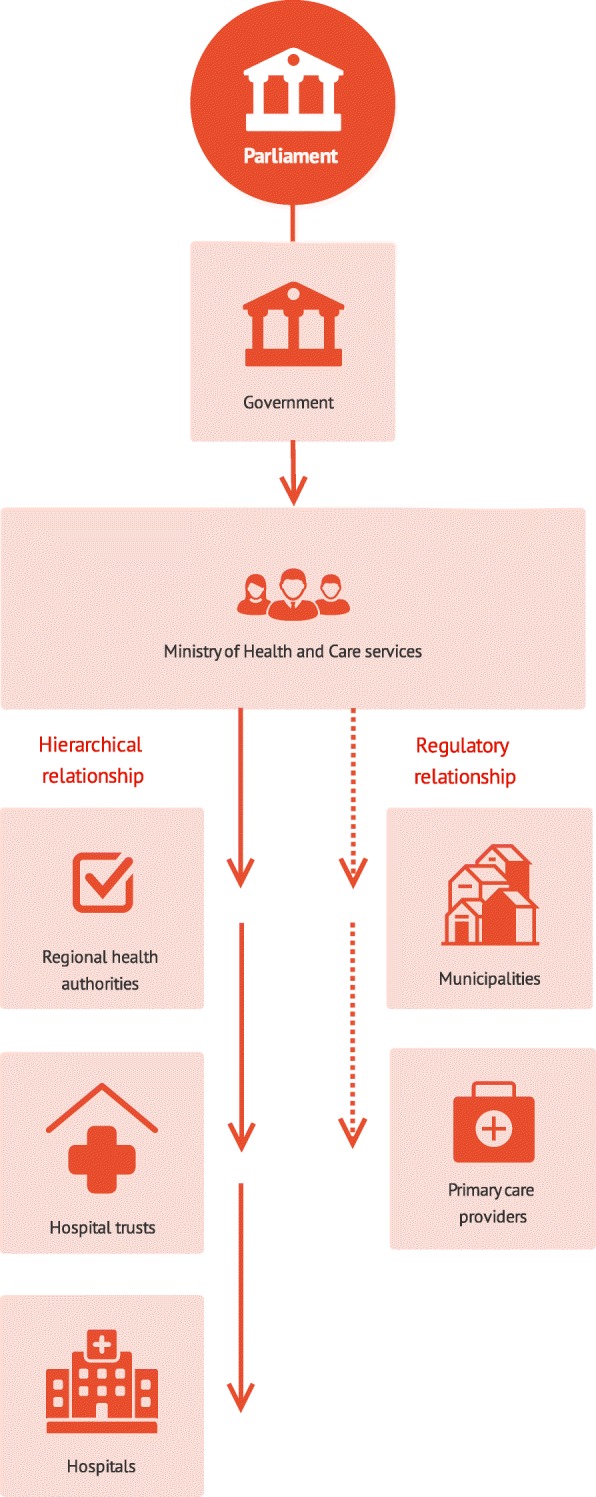
simplified model of the Norwegian health system based on Ringard et al. [43]

The Norwegian healthcare system is divided into primary and a secondary healthcare services (Fig. 2), each separately financed by public funds. The municipalities are responsible for providing primary healthcare services such as general practitioners (GPs), emergency rooms (ER), nursing homes and home care services, and each has different funding sources (municipal taxes, user fees and state grants) [18]. Apart from the earmarked subsidies provided by the state, the municipalities have discretion in how to organize and fund the primary healthcare services within the scope of overarching national regulations, leading to differences in how healthcare services are delivered between them (e.g. such as in nursing home coverage and staffing mix) [19].

The organization of care in the two included municipalities was similar. The nursing home services were divided into long-term- and short-term nursing homes. The short-term nursing homes were further divided into specialized wards like palliative care, rehabilitation, municipal emergency bed units (MEBUs) and hospital emergency bed units (HEBUs). MEBUs are placements for patients with conditions not requiring hospitalization where the municipality itself can examine, treat or provide care for the patient. HEBUs are beds for patients discharged from the hospital in need of further short-term treatment in the municipality [20]. The specialization of the short-term nursing home and the development of the MEBUs and the HEBUs came as a consequence of new demands imposed by the Coordination Reform. These reform measures also obligated the municipalities to co-finance municipal patients’ treatment in the secondary healthcare service, based on Diagnosis Related Groups (DRGs). Further, the municipalities have to pay the hospital costs for patients not in need of further hospital care. When the hospital physician assesses the patient as ready for discharge, the municipalities are contacted to provide further care within 24 h or pay the hospital per day fee of 4505 Norwegian kroner (USD$555) [21].

The nursing home physician coverage differed between the nursing homes and the municipalities. In the long-term nursing home in Municipality A, they had a regular physician present one day a week, while in Municipality B, three regular physicians were present four days a week. In both short-term nursing homes the physician was present five days a week. Common for all nursing homes was that the municipal emergency room (ER) doctor was responsible when the nursing home physician was not present during the day, afternoons, nights and weekends [25]. Apart from physician coverage, the total staffing per patient were similar but with some variation (Table 1). There were, however, larger differences in the distribution of nurses, certified nurse assistants (CNA), and assistants within and between the municipalities (Table 1).

**Table 1:** Overview of staffing-to-patient ratio

	Nursing home	Nurse to Patient ratio	Certified Nurse assistant to Patient ratio	Assistant to Patient ratio	Total healthcare worker to patient ratio
Municipality A	Short-term	1.09:1	0.42:1	0.18:1	1.69:1
	Long-term	0.45:1	0.96:1	0.12:1	1.54:1
Municipality B	Short-term	1.18:1	0.56:1	0.61:1	2.36:1
	Long-term	0.46:1	0.79:1	0.52:1	1.82:1

### Sample and recruitment of municipalities

Municipality A and B were recruited based on readmission rates reported by the Norwegian Directorate of Health in 2014 [22]. Once municipalities were identified, the head of the care department in each was approached and asked to provide contact information for suitable nursing homes and nursing home leaders. The nursing homes needed to be located within the included municipalities, and the leaders were selected based on their position in the included nursing homes. Further contact with the nursing homes was made by the first author. One short-term- and one long-term nursing home in each municipality was included in the study. The nursing homes were similar in organization but had differences in size and structure (Table 2). In cooperation with the first author, the recruited nursing home leaders invited nurses in their departments to participate in focus group interviews and scheduled the interviews based on the nurses’ working schedule. To be eligible for inclusion in the study, the nurses needed to work in a 50% position or more and have daily patient contact.

**Table 2:** Organization and structure of included nursing homes

Municipality A			Municipality B	
Nursing home	Short-term	Long-term	Short-term	Long-term
Beds	33	31	60	69
Wards	Palliative care, Municipal emergency bed units (MEBU), Hospital emergency bed units (HEBU), short term placements	Dementia and somatic wards	Palliative care, Municipal emergency bed units (MEBU), Hospital emergency bed units (HEBU), short term placements, rehabilitation	Dementia, somatic wards, short term bed
Total nursing homes in the municipalities	7		5	

### Data collection

Data collection consisted of semi-structured interviews with nursing home leaders and focus group interviews with nurses. One nursing home leader and one ward manager from each nursing home participated in interviews, with the exception of the long-term nursing home in Municipality B, where only the leader was available, making a total of seven leaders. Participants had variable levels of experience as leaders, but all had previously worked as nurses. Four focus groups (one for each nursing home) were conducted with groups of 3–6 nurses, resulting in a total of 17 participants. The nurses had differences in total work experience, work experience at the current nursing home and in their specialized backgrounds.

The interviews were conducted from September–October 2017. The focus group interviews with nurses were based on an interview guide (Additional file 1) with questions related to: *available resources and patient care; organizational structure and patient safety; changes in the organization;* and *implications of the Coordination Reform.* One moderator (first author) and one observer (third author) with healthcare backgrounds were responsible for conducting the focus group interviews. The interviews took place in the current nursing homes and lasted for approximately one hour.

Semi structured interviews with the leaders were conducted from October–November 2017 by the first author and covered similar, but slightly different topics to the nurse focus groups (Additional file 2), including: *available resources and finances; organizational structure and readmissions; changes in the organization;* and *implication of the Coordination Reform.* The interviews were conducted in the current nursing homes and lasted for approximately one hour. All interviews were audio recorded and transcribed by the first author.

### Data analysis

The data material was analyzed using Granheim and Lundmans’ qualitative content analysis approach [23, 24]. The first author (MKG) was responsible for the analysis with input from SW, TK, and OR who read transcripts and discussed theme development throughout the analysis period. JB and KC took part in discussions regarding theme development and refinement, and offered advice. Within-case analysis in each municipality was conducted. The embedded units (nurses and leaders in each nursing home) [25] were analyzed separately in accordance with Graneheim and Lundmans’ approach, to capture information within each unit [23]. Meaning components were extracted from the text and further condensed, coded and categorized with a focus on the manifest content [23, 24]. The categories from each embedded unit were later pooled together based on common characteristics and sorted under four sub-themes in each municipality showing small differences between the municipalities. An example of the analysis process in theme 1 is showed in Table 3. Next, the eight sub-themes were sorted under four common themes for the two municipalities with focus on similar features (Table 4). A cross-case analysis of the categories in each municipality was also conducted to find similarities and differences between the two municipalities [17]. These similarities and differences are considered in the results.

**Table 3:** Content analysis, within analysis, municipality A and B. Theme 1

Theme	Sub-themes	Category	Sub-category
T1: High nursing demands – variation in staffing and competence	Disparity in staffing and competence (Municipality A)	Tired nurses (NLT)	*Increased workload leading to de-prioritizing of important tasks*
		Complex patients (NLT)	*Seriously ill patients in need for complex treatment*
			*Elderly patients are not prioritized*
			*The patients don’t need to be hospitalized as often as before*
		Insufficient staffing (LLT + NLT)	*RN staffing not satisfactory - RNs confer with each other by telephone*
			*More nurses are needed, but there has been a staff reduction which increases sick leave among nurses*
			*Low physician coverage*
			*Trying to keep the balance between resource usage and patient safety*
			*Sicker patients gives new challenges*
		Acceptable staffing (LST)	*RN staffing is satisfactory in most wards*
			*Physician coverage is satisfactory*
		Unpredictable staffing (NST)	*Good physician coverage during weekdays*
			*There is not always enough RN staffing during weekends*
		Varying competence (LLT)	*Nurse competence is varying, but there is a focus on increasing it*
			*High demands in care leads to high demands in competence*
			*Fulltime positions is a way to increase competence*
			*To many assistants*
			*Reorganization can be positive in regards to competence*
		Sufficient competence (LST)	*The competence is high and have increased – increasing competence is encouraged*
			*The patient group makes need for high competence*
		High competence (NST)	*RN competence and staffing are high, but at the expense of LPNs*
		Capacity building in focus (NLT)	*Working towards increased competence*
	Reasonable staffing and competence (Municipality B)	Satisfactory staffing (LST)	*Staffing is satisfactory, but vulnerable during weekends*
			*Good physician coverage and cooperation*
			*RN density is increasing, which is positive for further recruitment*
		Acceptable staffing (LLT, NLT)	*The staffing is good as long as there is no sick leave*
			*There is a lot of assistants in some wards*
			*Competence is good, but capacity building on all stages are in focus*
			*There is a need for more LPNs*
		Sufficient competence (LLT)	*RN coverage and RN competence is high in accordance to more complicated patients*
		Satisfactory competence and staffing (NST)	*Physician coverage is good*
			*Nurse competence is varying, but several have continued educations*
			*There is focus on capacity building on all levels*
		Varying competence (LST	*Capacity building is in focus*
		Capacity building in focus (LST, NLT)	*Courses and guidance are not always provided but capacity building is organized internally*
			*The nursing home patients are more complex and sicker when discharged from the hospital*
		Internally organized capacity building (NST)	*Frustration in regard to discharge routines*
		Complex patients (LLT, NLT)	*The nursing home patients are more complex and sicker when discharged from the hospital*
			*The patients are more complex*
			*Frustration in regard to discharge routines*

**Table 4:** Overview of identified themes

Common Themes	T1: High nursing demands – variation in staffing and competence		T2: Disparate Perceptions of Organizational Conditions		T3: Economic limitations		T4: Perceived Predictors of hospital readmissions	
Sub-Themes	ST1: Disparity in staffing and competence (Municipality A)	ST1: Reasonable staffing and competence (Municipality B)	ST2: Organizational challenges on several levels (Municipality A)	ST2: Well-functioning organization on macro levels, challenges on micro levels (Municipality B)	ST3: Economic limitations do not affect access to medical equipment (A)	ST3: Opposing thoughts on economic limitations (Municipality B)	ST4: Predictors of hospital readmissions as perceived by nurses and leaders (Municipality A)	ST4: Predictors of hospital readmissions as perceived by nurses and leaders (Municipality B)

## Results

The analysis of the data material resulted in four common themes for both municipalities describing staffing and competence, organization and cooperation, economic factors and perceived predictors of hospital readmissions (Table 4). Variation and similarities were found across nursing homes and municipalities. Results below are reported following common themes for the municipalities.

### T1: High nursing demands – Variation in staffing and competence

#### Complex patients

There was a mutual agreement among nurses and leaders in both municipalities that the nursing home patients had become sicker and more complex following the Coordination Reform. A nurse in the short-term nursing home (STNH) of Municipality A described the patients as having “one foot in the grave” when receiving nursing home placement. Nurses in the long-term nursing home (LTNH) in the same municipality talked about complex nursing procedures and multi-morbid patients and that long-term patients were not necessarily long-term patients anymore because they often passed away a short time after arrival. Their leaders commented on this too:

*(…) We get new patients, they live for a month, and then there is new patients and then they live…well, they live for a very short time [after arrival]. And the month that they are here [at the nursing home], they require an extreme amount of follow-up. *(Leader LTNH, Municipality A)

In Municipality B, the nurses in the LTNH described their nursing home as a “small hospital” with advanced procedures and advanced patients. Their leader emphasized the complexity of the patients and described their conditions as being more time-consuming than before.

Nurses and leaders in both municipalities had a perception that patients were being discharged too early, and without completed treatment. Participants questioned whether patients were actually ready for hospital discharge due to the severity of their condition, especially in the STNH in Municipality B. In the LTNH of Municipality A, they had a perception that the hospital disclaimed all responsibility when they perceived the patient as ready for discharge. The nurses and leaders of the STNH in Municipality B also had thoughts about the complexity of the patients *they* discharged to the home care service.

*We got an admission to the MEBU. A lady with malnutrition and dehydration. When she came here, she both ate and drank herself and didn’t need any fluid treatment. She just needed the care (…) She thrived at the MEBU and was flourishing. Then she got discharged, and we were thinking, we need to be a little bit on the alert in this case. So we notified the need for close nutritional follow-up to the home care service. A week passed, and the patient was readmitted here at the ward, malnourished and dehydrated *(nurse STNH, Municipality B).

#### Capacity building

Capacity-building was a focus in both municipalities. In the LTNH in municipality A, capacity building involved educating nurses and encouraging assistants to take on formal education to become certified nurse assistants (CNA). There was variation across nursing homes in Municipality A as to whether leaders were the driving force in capacity building or if nurses themselves took responsibility such as by attending courses or starting continuing educations. Nurses in Municipality B described mostly taking responsibility for their own capacity building by learning from each other and seeking external competence (e.g. from hospital nurses within relevant wards) when necessary. However, the leaders in both nursing homes in Municipality B talked about having current formalized capacity building plans. In the long-term nursing home their plan revolved around increasing assistant competence, in the STNH they wanted to educate nurses within certain areas, such as palliative care or rehabilitation.

Nurses in both municipalities saw the distribution of CNAs and assistants as unsatisfactory. However, nurses in the LTNH of Municipality B described solving these problems by educating the assistants internally during lunch breaks and during every day work.

#### Competence

In Municipality A, the nurses of the STNH reported that the nursing home competence was high, and they found security in “always having someone to ask”. The leaders also agreed that competence was high; they perceived the nursing home to have one of the highest competence levels in the municipality. Leaders in the LTNH had experienced an increased need for nurse competence over the past six years, as a consequence of changes in the patient groups. They were still trying to meet this change in demand by increasing already existing nurse positions or changing CNA- positions in to nurse positions. The current competence of the existing nurses was described as varying.

*It’s people – like in other societies there are variations (…) some are fierce and seek new knowledge constantly, while others are at work, and then they go home… and… it varies. *(leader STNH, Municipality, A).

In municipality B the leaders of the STNH described the nurses’ competence to be varying, yet most of the employed nurses had completed one or several continuing education programs within relevant areas. The leader of the LTNH described the competence to be good on all levels and strongly believed that the patients were being well taken care of.

#### Staffing

There were variations in perceptions of staffing between and within municipalities. In Municipality A, in the fall of 2016 they had received instructions from the municipality’s administrators to cut staffing. Leaders in the STNH stated that the overall staffing was seen as reasonable to cover the needs of the patients despite these cuts. The nurses were, however, dissatisfied with the staffing levels during weekends, because they were sometimes unpredictable as nurses on sick leave were replaced by assistants. The leaders supported this claim and described the nurse coverage as satisfactory in some, but not all, wards. They further described limitations in hiring in extra people for patients needing one-to-one supervision.

*Your gut feeling tells you that this is not okay. There should have been more personnel, but you are feeling the pressure from both the leader and the municipality; that we should manage without hiring extra people in … that can be hard sometimes *(leader STNH, Municipality A).

The leaders of the LTNH in Municipality A also indicated that they still managed to run the nursing home in a professional manner with the current staffing levels, but the recent staff cuts affected the nurses negatively and the proportion of sick leave had increased, putting even more pressure on the remaining nurses. One of the nurses commented on this too:

*(…) I am working my way out of the ward. I’ve taken out the trash, I’ve wiped, and then I’m supposed to do the administrative work … so I work like a horse. I am totally beat when I get home. I’m starting to feel that this isn’t right! It shouldn’t be like this at a work place *(nurse LTNH, Municipality A).

The nurses further experienced an increased amount of administrative work after a change in the leadership structure of the nursing home. They also often reported being the only nurse during a shift, causing even more pressure and insecurity. The leaders confirmed these problems and were constantly trying to balance finances and quality of care.

In Municipality B the nurse staffing levels were described as satisfactory in both nursing homes most of the time, but vulnerable during weekends or if nurses were on sick leave. The leader of the LTNH was still working on increasing the nurse coverage and, even though recruitment had become easier after the increased nurse density in the nursing home, there were still some struggles in attracting new nurses.

#### Physician coverage

The physician coverage was reported as high in the short-term nursing homes of both municipalities. In the LTNHs there was some variation. In Municipality A, leaders expressed the view that physician coverage was too low, with a physician only present a few hours a week. The leader of the LTNH in Municipality B suggested it would be advantageous to have a physician present 24 h a day, 7 days a week, but still found the physician coverage sufficient to carry out a proper follow-up of the patients.

*In terms of planned interventions, the patient follow-ups and the regular tasks, there is enough hours [physician coverage] in my opinion. But it would have been all right to have a physician available at all times… because there are those things that happen right after the physician leaves… *(leader, LTNH, Municipality B).

There was an overall satisfaction with the cooperation between the nurses and the nursing home physicians in all nursing homes. However, there were reports of difficulties in cooperation with the emergency room physicians in both municipalities.

### T2: Disparate perceptions of organizational conditions

#### Inadequate admission routines

Several organizational difficulties were described in the STNH in both municipalities. In the MEBUs, there were descriptions of patients sometimes being admitted by general practitioners (GPs) or ER doctors without having been clinically assessed or diagnosed by the physician beforehand. In Municipality B they further experienced patients in need of hospital care being wrongfully admitted to the MEBU, and then transferred to the hospital a short time after arrival.

In Municipality A there were also reports of patients arriving from the ER without the nurses being notified ahead of time or this information not reaching the nurses at the moment of arrival. The time of arrival from the hospital was sometimes seen as inappropriate.

*It is fun [ironically] when they arrive [from the hospital] a Friday night and there is no physician on call during weekends … Yeah, and on Monday they get readmitted to the hospital, or the next day, on Saturday. *(nurses STNH, Municipality A).

#### Patient flow and patient composition

The patient flow was experienced as an organizational challenge by the leaders of the STNH in Municipality A. They were having problems discharging patients in need of further care due to lack of nursing home placements, causing a bottleneck situation. Consequently, MEBUs, which are a 72-h offer, were filled up, occupying beds for patients in acute need of care placement. In Municipality B, they had experienced pressure to discharge patients out of the short-term placements due to full wards. Further, the patient composition was sometimes seen as a challenge in the MEBUs in both municipalities:

*We are a reception ward [MEBU] and are supposed to take care of all kinds of patients. That can be a challenge sometimes. Patients with disruptive behavior inside a ward where… where people [have] chronic obstructive pulmonary disease (COPD) for example. People are terminal, they’re having panic attacks, and they hear people in the hallway screaming and shouting and … yeah … yelling at the staff. *(leader STNH, Municipality A).

#### Organization and cooperation

The STNH of Municipality A was struggling with saving measures introduced by the municipal leaders, where single rooms were converted to double rooms, leaving an entire ward empty. With all patients gathered in one ward, less personnel were needed to take care of the patients. The nurses described it as cramped as there were now two patients in a room originally designed for one. If a patient arrived who was unable to stay in a double room, or if two patients did not get on well together, this involved a lot of moving of the patients to find suitable solutions.

In Municipality B, there were different opinions and focuses around the organization in the two nursing homes. Firstly, leaders in both nursing homes found co-location of short-term and long-term services positive. The leader of the STNH highlighted the stability the patients experienced when they were coming to the same place every time they needed a short-term stay. The leader of the LTNH suggested that the managing of one service led them to being more focused and effective and the services becoming more specialized. The leader was, however, not satisfied with the new cooperation agreement between the health trust and municipalities developed after the Coordination Reform and claimed that some guidelines imposed by the state went against their previous agreement, making the coordination between the services more challenging. However, the nurses in the LTNH described the cooperation between the hospital and nursing homes when the patients were discharged as good.

*I remember last time, when we were learning how to use the BiPAP* [Bi level positive airways pressure] *machine. The patient was hospitalized and they were waiting to discharge the patient until we let them know that we’ve had training in using the machine… It helps when they give us time *(nurse LTNH, Municipality B).

### T3: Economic limitations

Access to medical equipment did not seem to be an issue affecting hospital readmissions in Municipality A. Although leaders in both nursing homes reported financial limitations, access to medical equipment was perceived as sufficient. Leaders in the LTNH admitted that the equipment budget could have been larger, but they managed to buy what they needed and invested in equipment where necessary. The leaders in the STNH of Municipality A, reported having “plenty” of medical equipment after the start of a new patient safety project. The financial costs of this equipment were covered by grants that went beyond the usual budget as it was linked to a patient safety project.

In Municipality B, the leaders agreed that economic factors and the need for cost savings had always been a common issue in primary care.

*Finances and professional soundness are always a theme and a dilemma. To find a resource use that secures a good night’s sleep and at the same time doesn’t cost so much that it destroys your sleep, I guess that is what it is all about *(leader LTNH, Municipality B).

Leaders in the STNH of Municipality B stated that the financial situation was improving and that saving measures most commonly affected the staffs’ access to external courses and did not affect the patient care or the equipment needed to provide proper care. The nurses in the same nursing home disagreed with this perspective and reported that economic factors in fact did affect the patients’ access to medical equipment such as bandages. Some nurses further suggested that money was prioritised over patients’ needs and that they were told to give the patients good care, but not the best care.

*In this job, I early on sensed that the nursing profession and the healthcare services’ economy often are in conflict. And I am realizing how fast we are adapting to the economic constraints. We become very obedient to the organization, and sometimes we are in conflict with ourselves and we are contradicting our own professional assessments based on the economy we have … *(nurse STNH, Municipality B).

The leader of the LTNH in Municipality B compared access to technical helping aids with EHS (Environment, Health and Safety), and indicated that helping aids were ordered as needed to avoid sick leave. The nurses in the LTNH were satisfied with the access to equipment.

### T4: Perceived predictors of hospital readmissions

Nurses and leaders within the LTNH, in both municipalities, reported that there was an overall focus in avoiding hospital admissions and readmissions and that hospital readmissions were seen as highly necessary when they occurred.

A number of factors were perceived as influencing readmissions in the two municipalities. First, high nurse competence in the nursing homes was stated as an important aspect in avoiding the need for hospital readmissions

*I believe that competence is alpha omega [fundamental] in avoiding hospital readmissions. The more competence we have, the more [patients] we can take care of here [in the nursing home]. And we have the equipment to do so! *(leader LTNH, Municipality A).

The leader further suggested that if the nurses were unsure of their ability to handle complex patients, it could lead to hospital readmissions. In Municipality B, nurse staffing was mentioned as another important factor. The leader of the LTNH expressed that it was of importance that the nurses had nurse colleagues to discuss daily issues with; the threshold for contacting the ER doctor if problems occurred became higher when there was room for professional discussions between nurses.

Physician coverage and physician competence was seen as important in both municipalities. In the LTNH of Municipality B the nurses stressed the importance of having a physician present, as they had most days of the week. Their leader further suggested that the more often a physician was present at the nursing home, the more his or her knowledge of the patients would increase, as would the possibility of having a good dialogue with the patients’ next of kin, which could lead to the prevention of hospital readmissions. In Municipality A, they emphasized good communication between the physicians in the municipality as a key factor. Participants suggested that the nursing home physicians should provide and document clear guidelines for the treatment of the nursing home patients so that other physicians involved in the patient treatment could access this information easily. In Municipality B, they talked about the importance of good cooperation between the health service agencies in general. More interdisciplinary cooperation was also mentioned as a factor preventing hospital readmissions.

*When I started to work here last year, we had physiotherapist and an occupational therapist present every day. We sensed that this was a good tool in preparing the patient for discharge. Now, we only have them here a couple of hours a week, and that is very little. Things are being discontinued in regards to rehabilitation and preparation for discharge and in preventing hospital readmissions *(nurse STNH, Municipality B).

Nurses and leaders in both municipalities believed that longer hospital stays were important in preventing hospital readmissions. Further, the leaders in the STNH of Municipality A strongly believed that the care they provided was a preventer of hospital readmissions.

*If they [patients] had been sent home straight after [a hospital stay], they wouldn’t have had the strength to, in a way, do that much, they would have been in a greater risk of being readmitted if they were to come home [instead of STNH] *(leader STNH, Municipality A).

Pressure from the patient’s family were, in the STNH of Municipality B, seen as a factor leading to hospital readmissions. If the patients’ family put a lot of pressure on getting the patient admitted to the hospital, for example, because they believed that the patient could get better treatment there, it was hard to resist.

Lastly, the leader of the LTNH in the same municipality reported that physical distance to hospital affected hospital readmissions. If the nursing home was closer to the hospital, it would be easier to admit a patient to be “sure”, while it would be a much bigger constraint for the patient if the hospital was further away. The leader also mentioned the media and the attention of the media on all the things going wrong in the healthcare service, which was putting pressure on the physician in charge in deciding whether to admit or not.

## Discussion

From the nurses’ and leaders’ perspective, the nursing homes patients had become sicker and more complex after the introduction of the Coordination Reform, consequently demanding greater nursing competence. The access to resources in terms of staffing, competence and physician coverage varied across nursing homes, however, capacity building was an overall focus. Organizational difficulties were mostly detected in the short-term nursing homes, but organizational changes had also had positive effects. As seen by the nurses and leaders, economic limitations and municipal attempts at cost savings were present, but did mostly not affect patient care and the ability to access medical equipment. Several factors were perceived as affecting hospital readmission by nurses and leaders (e.g. nurse staffing, competence, physician coverage, nursing home documentation, early hospital discharges). All of this concertedly influences measures which have to be considered to prevent hospital readmissions.

### Nurse staffing and physician coverage

In all nursing homes, there were descriptions of increasing complexity in the care needs of nursing home residents, forcing the nursing homes to function as small hospitals. This increase in complexity has previously been described [6, 25], and was stated as a natural development in accordance with the new directions of the Norwegian Coordination Reform [26]. The acuity of the patients has been reported to be too high in previous research, making hospital admissions or readmissions necessary due to the lack of adequate nurse competence or resources [27]. Nurses and nursing home leaders in the current study described caring for patients who had become so deteriorated when arriving to the LTNH that the long-term care function had shifted towards a short term acute function, with high mortality rate of admitted patients. These findings are supported by a recent Norwegian study showing an increase in mortality among nursing home patients since the introduction of the Coordination Reform [28]. Despite this shift in the patient group, the LTNHs in this study had lower physician and nurse coverage than the STNH, and one could question if these new conditions had not been taken into consideration by municipal leaders. Previous research has shown that unstable nurse coverage and low nurse staffing in nursing homes is a factor increasing the propensity for hospital admissions and readmissions [29, 30]. A previous study, within the two case municipalities, showed that extensive use of ER doctors was another factor increasing the chance of hospital readmissions, as the ER doctors were not familiar with the patients and their clinical status [27]. Higher levels of continuity of care in doctors have also proven to be associated with lower mortality rates [31]. Previous research demonstrated connections between low nurse staffing and direct/indirect adverse patient events in hospitals [32]. There is, however, little knowledge about staffing and adverse events in the municipal healthcare service, but the results here support the view that unsatisfactory nurse staffing can lead to greater risk of adverse events and, further, hospital admissions and readmissions. With that in mind, the overall aim in reducing hospital readmissions [33] is at odds with current physician and nurse staffing levels in the LTNH, unstable weekend nurse staffing across nursing homes and the recent staff cuts experienced, particularly in Municipality A.

### Competence and capacity-building

Capacity-building was a focus in all nursing homes, but competence seemed to mean different things to the participants of the study. Where the nurse staffing was perceived as unsatisfactory, competence first of all meant increasing the nurse coverage. In all nursing homes competence meant increasing the number of CNAs and decreasing the numbers of assistants. Another overall understanding of competence was to have nurses with continuing education (e.g. geriatric nurses or wound nurses). The nurses in the STNH of Municipality B reported desiring more competence from other fields of expertise (e.g. physiotherapists and occupational therapists). These nurses had one or several continuing education programs, which could indicate varying focuses in capacity building depending on current competence holdings in the nursing home. Variation in nurse competence [34] and unsatisfactory competence in nursing homes [27] have also been recognized in previous research. The participants in the current study (nurses and leaders) and the participants in a previous study (GPs and nursing home physicians) [27] had an overall belief that nurse competence in the municipal healthcare service was a crucial factor in reducing hospital readmissions. Romøren at al. (2017) further showed that organized training in specific nurse procedures (intra venous antibiotic treatment) in nursing homes increased the nursing home’s ability to care for the patients onsite [35].

### Nursing home organization

Organizational difficulties were most often reported by participants from the STNHs. In both municipalities there were issues associated with the admission process to MEBUs (e.g. undiagnosed patients, missing treatment plan, wrongly placed patients, time of admission) which could lead to hospital readmissions. These difficulties can be connected to the novelty of the MEBU institution, and may have been caused by routines not being inculcated among the physician population, or unclear rules in the application of the beds [36]. A report describing the function of the MEBUs also shows that the criteria for admitting patients to the MEBUs are inconclusive and highly subjective, making it difficult for the physicians to make accurate decisions [37]. Lastly, these difficulties can be a result of understaffed or busy ERs with limited time to do the necessary preparatory work before admitting patients to MEBUs [25]. Another organizational problem described in the MEBUs (a 72-h stay) manifested in the difficulties in providing further care for patients in need of long or short-term institutional care after their stay. Problems in providing nursing home placements and the negative effect this has on hospital readmissions have been described elsewhere [25, 38].

### Human factors and system perspective

**Fig. 3:**
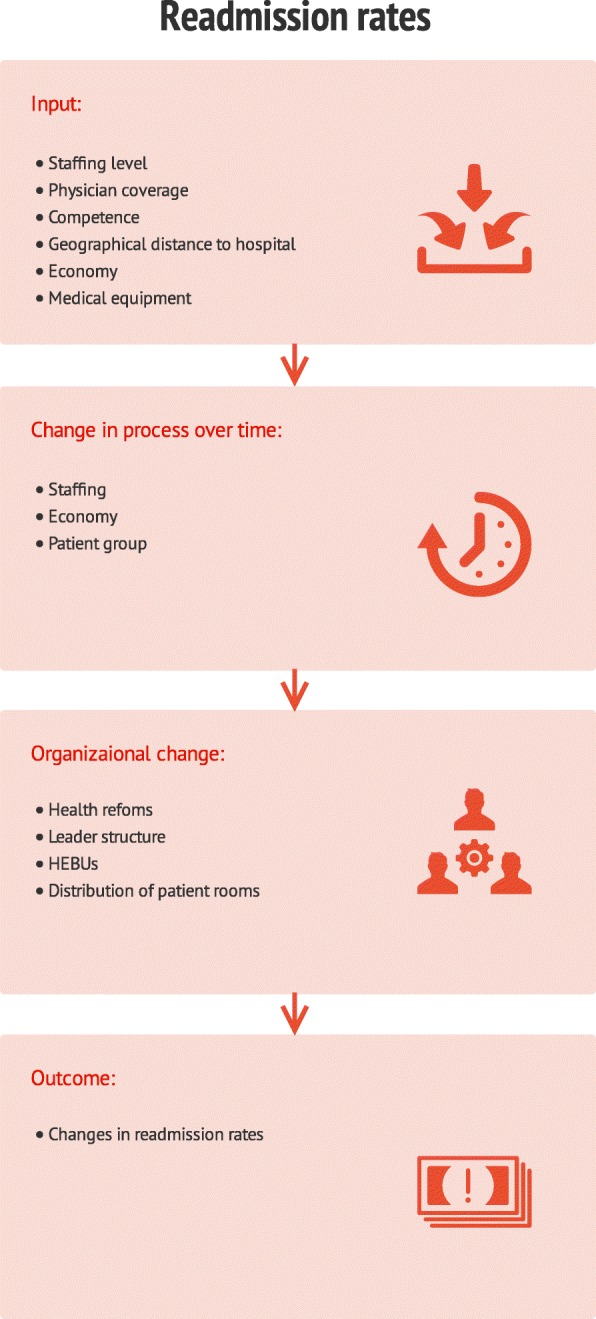
Integrated model to understand factors contributing in affecting hospital readmission rates

Overall, our study shows that nursing home leaders and nurses perceive multiple factors contributing to work practice in nursing homes and decisions about whether a patient can be treated in a nursing home or if a hospital readmission is required. Our results can be interpreted using a human factors and systems perspective, which focuses on the interactions between the environment and the individuals within it [39]. Healthcare personnel have tasks that need to be conducted with the help of different tools or instruments such as hospital stay summaries or appropriate medical equipment. These tasks are conducted within a specific physical environment under specific organizational conditions, which in our study include increasingly complex patients, reduced capacity (staffing, competence, beds) to receive patients and sometimes limited access to physicians. Five components of a human factors perspective as described by Carayon et al. [39]—person, task, tool/instrument, physical environment, and organizational conditions—all interact with, and affect, each other, and produce diverse outcomes such as performance, safety, work life, quality and health. All parts in an organization affect and depend on one another. Changes, such as the Coordination Reform or cuts in staffing, can therefore affect organizational outcomes [39]. More specifically, a human factors perspective can be suitable for understanding the organization and context of primary healthcare services and how this affects variation in readmission rates [40]. Our study shows that the readmission rates have evened out during the period of study and factors that might explain why, relate to competence level, staffing level, physician coverage, time of discharge from hospital, interaction between hospital and nursing home, and nursing home organization (Fig. 3).

### Strengths and limitations of the study

This study is the first of its kind to explore nurses’ and nursing home leaders’ experience of the resource situation, staffing and competence level in relation to the readmission issue from the perspective of nursing home nurses and leaders, and particularly in the context of a major reform to primary and hospital services (the Coordination Reform). As to the limitations, rather than demonstrating causation, the study provides insight into the possible associations between variables. The study also consisted of a small sample of staff and nursing homes in two municipalities limiting the scope and generalizability [41]. This was particularly true in LTNH in Municipality B, where despite efforts they were only able to recruit one leader. Nevertheless, it provides an in-depth insight into the selected municipalities and establishes a logic that could be applicable to other similar contexts [42].

Other limitations relate to methods of recruitment and data collection. The nurses included in the study were selected by their leader, with the risk of selection bias or unintended pressure to participate in the study. All the participants were, however, informed about their right to withdraw from the study at any time. Nurse selection by leaders was necessary to combine interview time with work schedules and staffing in the wards. Focus group interviews may introduce bias related to group dynamics [41], for example, that participants have different degrees of introversion and extroversion, making some opinions potentially more dominant than others. This was taken into consideration during the focus group interviews by directly asking questions to “silent participants”.

The overview of staffing to patient ratio in the nursing homes (Table 1) are based on the total amount of employees in the nursing homes. This table do not provide information about the ratio on each shift, vacant positions, part time positions, patient overlay or unoccupied beds, and can therefore not provide a complete picture of the staffing situation.

### Implications for practise and further research

This paper provides an insight into nursing home staff’s perspectives on resource situation, staffing and competence level and factors affecting hospital readmissions, and suggests measures that could prevent hospital readmissions (e.g. improved documentation in the nursing homes, adequate staffing and competence, better coordination between the health service levels). The paper further shows that there is a complex interaction between the constituent factors in hospital readmissions, which is not yet fully understood, demonstrating a need for further research. More research on readmissions from a municipal or primary care perspective, involving the home care service and the hospital physicians’ experience of readmissions from the municipalities is needed.

## Conclusions

The findings suggest that nurses and leaders experienced an increasingly complex patient group which required improved competence in the nursing homes. In line with the changed patient group, patients had become more demanding and mortality among nursing home residents were perceived to have increased, producing a shift in the long-term nursing homes towards short term institutions and forcing short term nursing homes to function as small hospitals. Staffing, competence and physician coverage did not seem adjusted to the new demands in all the nursing homes. Access to medical equipment was seen as satisfactory, and patients were mostly not affected by ongoing attempts at savings in the municipalities. Several factors were suggested to affect hospital readmissions (e.g. high nurse competence, nurse and physician staffing, early hospital discharge) and the municipalities were similar in their answers regarding the importance of these factors. The cross-case analysis showed differences in nurse staffing and physician coverage and that capacity building had different meanings in the institutions, depending on their resource situation.

## Additional files

Additional file 1: Interview guide, focus group interview, nurses in nursing homes. (DOCX 15 kb)

Additional file 2: Interview guide, individual interviews, nursing home leaders. (DOCX 15 kb)

## Abbreviations

DRG: Diagnosis related groups
EHS: Environment, health and safety
ER: Emergency room
GP: General practitioner
HEBU: Hospital emergency bed unit
LLT: Leaders long-term
LPN: Licenced practical nurse
LST: Leaders short-term
LTNH: Long-term nursing home
NLT: Nurses long-term
NST: Nurses short-term
STNH: Short-term nursing home
