# Supplementary material for: Nursing home leaders’ and nurses’ experiences of resources, staffing and competence levels and the relation to hospital readmissions – a case study
Source: BMC Health Serv Res. 2018 Dec 12;18:955. doi: 10.1186/s12913-018-3769-3 (PMC6292004; doi:10.1186/s12913-018-3769-3)
Supplement: Supplementary file 1 — Interview guide, focus group interview, nurses in nursing homes. (DOCX 15 kb) [file 12913_2018_3769_MOESM1_ESM.docx]

Interview guide – nurses (focus group)

**Introduction**

- Aim of the study
- Use and storage of data
- Anonymity and confidentiality
- Endurance and structure of the interview
- Why focus group interview
- Consent to record the interview

**Background information**

- Previous work experience
- Experience as nurse
- Experience in current position

*Each member of the focus group introduce themselves with name, which ward they are working in, how long they have worked as a nurse, where they worked before and how long they have worked in the current work place.*

*Present: Moderator and observer*

**Changes in the organization of the institution – Coordination reform**

1. How would you describe the patient group that you are working with? Has it changed since the introduction of the Coordination reform?

| **Additional questions:**  Can you describe any changes in the patients’ illnesses, age, function level |
| --- |

1. Can you tell us about any changes in the organization of the nursing home that have affected your work as a nurse in one or the other way?

| **Additional questions:**  Have these changes happened as a consequence of the Coordination reform? In what way? |
| --- |

**Available patient resources and nurses**

1. Can you tell us about a situation where you have felt unsafe or unsecure in the treatment of a patient related to lack of competence? What was the reason?
2. Can you tell us about how you, as nurses, through your work place, can become better prepared to care for the patients in all situations?

| **Additional questions:**  How do you experience that your competence is good enough in the daily work with the patients?  What do you think about the availability in courses or educations?  How do you consider the current approach for capacity building among your leaders? |
| --- |

1. In what way do you experience that economy affects your daily work?

**Cooperation between physicians and nurses**

1. How do you cooperate with physicians / ER physicians when a hospital readmission is being considered?

| **Additional questions:**  What role do you play in questions of hospital readmissions?  Has this role changed? |
| --- |

1. In what way do you think you influence decisions in questions of hospital readmissions?
2. In what way do patients and next of kin influence you in questions of hospital readmissions?
3. Are there any tools you can use when you assess/report the patients’ condition to the physician? If so, can you tell a little bit about how it is used, and what you think the purpose of it is?

**Organizational structure and patient safety?**

1. Do you have any examples of situations where the patients’ safety were well taken care of in the nursing home? Any examples of the opposite? What was the reason?
2. Have there been any structural changes after the implementation of the Coordination reform? (Changes in competence, patient organization, staffing)? If so, how has this influenced the patient safety?
3. Based on your experience, why do you think there are differences in readmission rates between municipalities affiliated with the same hospital?

| **Additional questions:**  Do you think there are any specific reasons?  Can you think of any measures, which could have reduced hospital readmissions? |
| --- |

1. How do you think the current combination and organization of healthcare personnel in the different wards are working?

**Summary**

1. Is there anything you believe to be relevant to the topic, which has not been addressed in this interview?

- Review of the most important topics appearing during the interview.
- Clarify any potential misunderstandings.
- Additional comments?
