# Supplementary material for: Nursing home leaders’ and nurses’ experiences of resources, staffing and competence levels and the relation to hospital readmissions – a case study
Source: BMC Health Serv Res. 2018 Dec 12;18:955. doi: 10.1186/s12913-018-3769-3 (PMC6292004; doi:10.1186/s12913-018-3769-3)
Supplement: Supplementary file 2 — Interview guide, individual interviews, nursing home leaders. (DOCX 15 kb) [file 12913_2018_3769_MOESM2_ESM.docx]

Interview guide – nursing homes leaders

**Introduction:**

- Aim of the study
- Use and storage of data
- Anonymity and confidentiality
- Endurance and structure of the interview
- Consent to record the interview

**Background information:**

- Work experience
- Experience as a leader
- Short description of leader tasks in the current institution

**Resources and economy**

1. Can you tell me about the economic situation in the nursing home?

| **Additional questions:**  Have there been any changes in de financial situation in the nursing home after the implementation of the Coordination Reform? |
| --- |

1. What are your thoughts on the relationship between the availability of resources (room, equipment, competence etc.) and patient safety?

| **Additional questions:**  How do you assess the situation as it is here (at the nursing home) |
| --- |

1. How do you think resources influence hospital readmissions?
2. Can you tell about a situation where you have felt that economy or resources have put the patient’s safety at risk, if such a situation has taken place?
3. How do you experience the physician coverage and the nurse coverage at the nursing home? Is it sufficient?

| **Additional questions**:  Can you give any examples? |
| --- |

1. How do you experience the employees’ competence?
2. In what degree do you experience that the competence and staffing levels are sufficient in the nursing home?

| **Additional question**:  What are any additional needs? |
| --- |

**Organizational structure and hospital readmissions**

1. What do you think are common reasons for hospital readmissions from the nursing home?
2. There are differences in readmission rates between municipalities affiliated with the same hospital. What do you believe can be the reason for this?
3. Based on your experience, in what way do you think that the nursing home organization can reduce hospital readmissions?

| **Additional question:**  How do you experience compliance between the need for competence and capacity and current organization and resources? |
| --- |

**Changes in the organization – the Coordination reform**

1. Can you describe any changes in the organization made in relation to the introduction of the Coordination reform?

| **Additional questions:**  What changes in the organization of the nursing home or the ward done in relation to the Coordination reform do you experience as challenging? Why? |
| --- |

1. How has the Coordination reform influenced the quality of the healthcare services in your opinion?

| **Additional question:**  Has it been positive or negative?  Can you provide any examples?  Do you think this influence hospital readmissions? |
| --- |

**Summary**

- Review of the most important topics appearing during the interview.
- Clarify any potential misunderstandings.
- Additional comments?
